# Microbial Biofilm as a Smart Material

**DOI:** 10.3390/s150204229

**Published:** 2015-02-12

**Authors:** Christian Garde, Martin Welch, Jesper Ferkinghoff-Borg, Thomas Sams

**Affiliations:** 1 Center for Biological Sequence Analysis, Department of Systems Biology, Technical University of Denmark, Kemitorvet 208, DK-2800 Kongens Lyngby, Denmark; E-Mail: garde@cbs.dtu.dk; 2 Department of Biochemistry, University of Cambridge, Hopkins Building, Downing Site, Cambridge CB2 1QW, UK; E-Mail: mw240@cam.ac.uk; 3 Biotech Research & Innovation Centre, Faculty of Health and Medical Sciences, University of Copenhagen, Ole Maaløes Vej 5, DK-2200 Copenhagen N, Denmark; 4 Biomedical Engineering, Department of Electrical Engineering, Technical University of Denmark, Ørsteds Plads 349, DK-2800 Kongens Lyngby, Denmark

**Keywords:** quorum sensing, size sensor, AHL, PQS, *Pseudomonas aeruginosa*, OdDHL, signal molecule, filtering, switch, biofilm, 87.18.Fx, 87.18.Gh, 87.18.Cf, 87.18.Tt, 87.85.fk, 92C05, 92C37, 92C42

## Abstract

Microbial biofilm colonies will in many cases form a smart material capable of responding to external threats dependent on their size and internal state. The microbial community accordingly switches between passive, protective, or attack modes of action. In order to decide which strategy to employ, it is essential for the biofilm community to be able to sense its own size. The sensor designed to perform this task is termed a quorum sensor, since it only permits collective behaviour once a sufficiently large assembly of microbes have been established. The generic quorum sensor construct involves two genes, one coding for the production of a diffusible signal molecule and one coding for a regulator protein dedicated to sensing the signal molecules. A positive feedback in the signal molecule production sets a well-defined condition for switching into the collective mode. The activation of the regulator involves a slow dimerization, which allows low-pass filtering of the activation of the collective mode. Here, we review and combine the model components that form the basic quorum sensor in a number of Gram-negative bacteria, e.g., *Pseudomonas aeruginosa*.

## 1. Introduction

Bacteria are remarkable decision makers that are capable of responding to a multitude of environmental challenges through changes in gene expression [1]. Over the past twenty years, it has become increasingly evident that bacterial decision-making often exceeds the classical paradigm of individual, isolated cells [2–4]. Many Gram-negative bacteria form microbial communities denoted as biofilms, which confer tolerance to environmental stress and bactericides [5,6]. In addition to this passive benefit, the biofilm provides the bacteria with optimal conditions to employ a colony-size-dependent mode of gene regulation, which enables coordinated responses on a colony-wide scale. This regulatory system, denoted quorum sensing (QS), guides the bacteria to time the production of factors that are only favorable at high population densities [2,7].

The first observation of this collective sensing was made in the luminescent *Vibrio fischeri*, which populates the light organs of squids. QS ensures that visible light is only synthesized when a sufficient number of bacteria have amassed to meet the production requirement [2].

QS is also applied by pathogens to mount a successful infection. This is partially achieved by promoting biofilm formation, but also by arresting the production of immune-triggering virulence factors to evade the host defenses in a stealthy fashion until the bacterial colony has reached a size capable of withstanding the host response [8,9]. Furthermore, recent lines of evidence suggest that QS also confers immunomodulatory effects to limit the effectiveness of the host immune system [10].

A classical example is chronic infections by *P. aeruginosa* in the lungs of cystic fibrosis patients. Here, the interplay between the biofilm and QS regulation confer high drug tolerance and impairment of the immune system, rendering the infections almost impossible to eradicate within traditional treatment regimes [9].

In this paper, we review and combine the model components as we suggest they may look in the basic quorum sensors as observed in a number of Gram-negative bacteria, e.g., *P. aeruginosa* [11–14]. Our primary goal is to present a modeling perspective on the ingenious designs of the switches that are made possible using no more than two genetic components with a single positive feedback. We shall attempt to use as little mathematics as possible while still retaining the key features of the system.

## 2. The Reaction-Diffusion Equation

**Figure 1.**
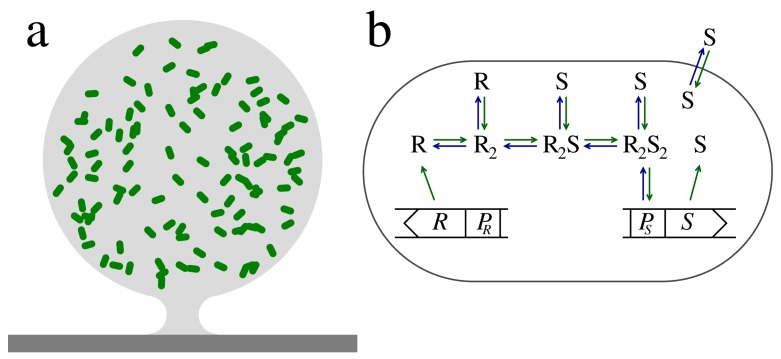
**(a)** Bacteria residing in a spherical biofilm micro-colony attached to a surface; **(b)** example of a regulatory motif in a single cell. The signal molecules S are synthesized under the control of the promoter *P_S_*. The signal molecules diffuse freely in/out of the cell and between cells in the colony. The cell produces a regulator, R, which dimerizes and binds signal molecules, typically from other cells, to form the activated regulator, R_2_S_2_. The concentration of the activated regulator now constitutes a proxy of the “size” of the population and can be used to control size-dependent gene regulation. The activated regulator can bind to the promoter to increase the signal molecule production. When this happens, the positive feedback ignites the whole colony, allowing for the concentration of signal molecules and activated regulators to increase dramatically. The figure is adapted from figures in [11]—published by The Royal Society of Chemistry.

Consider a spherical micro-colony of bacteria in a biofilm matrix of radius 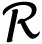 as shown in Figure 1. The production and diffusion of signal molecules with concentration *s* = [S] are linked by the diffusion equation:

(1) $$\frac{\partial s}{\partial t}=D\Delta s+\rho_{v}\kappa_{s}$$

The first term is the diffusion term, *i.e.*, *D* is the diffusion constant and Δ is the Laplacian operator. The second term is the production of signal molecules per volume written as the product of the intracellular production, *κ_s_*, and the volume fraction occupied by cells, *ρ_v_*. The signal molecules are assumed to diffuse freely through the cell membrane, and the diffusion constant in the biofilm matrix is similar to that in water.

## 3. Signal Molecule Sensor

A typical way for a cell to sense the presence of a molecular species, S, involves binding to a regulator protein, R, to form an activated RS-complex. In LuxRI homologues, which are frequently found in Gram-negative bacteria, the common motif appears to be that regulators dimerize and subsequently bind signal molecules [7,12,15–19]. Also, the PqsRI quorum sensor in *P. aeruginosa* produces a regulator that appears to be dimeric in its active form [14].

The concentration of the RS-complex is the intrinsic measure of the quorate level. The RS-complex can subsequently target specific promoter sites on the DNA to produce the desired response. In the quasistatic limit, the concentration of the activated regulator may be expressed as:

(2) $$r_{a}=\frac{s^{2}}{K^{2}+s^{2}}r_{m}$$

where *K* is an (effective) dissociation constant for binding of the signal molecules to the regulator. Equation (2) describes dimerization followed by cooperative binding of signal molecules [20]. Non-cooperative binding:

(3) $$r_{a}=\frac{s^{2}}{{\left(K+s\right)}^{2}}r_{m}$$

is also of interest, since it appears to be found for some quorum sensing signal molecules [14]. We shall go into some detail on the dimerization and ligand binding below, where we will also review the filtering permitted in these designs [12,13].

We note that, when there is negative feedback from the activated regulator to the regulator production, it can be hard to distinguish the behavior of the dimeric system from a monomer. This could account for the monomeric behavior for the ligand binding reported in *Aeromonas hydrophila* [13].

## 4. Signal Molecule Production and Feedback Ignition Point

**Figure 2.**
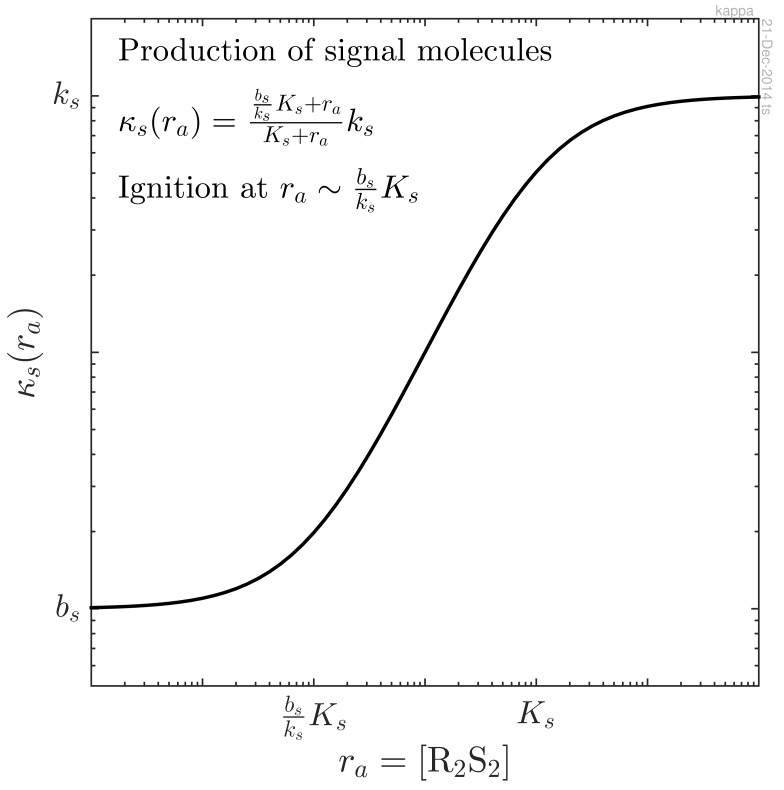
Log-log plot of signal molecule production, *κ_s_*, as a function of activated regulator concentration, *r_a_* = [R_2_S_2_]. The feedback loop ignites when the signal molecule production starts rising from the background level. Note that this point can be several orders of magnitude lower than the dissociation constant for the binding of R_2_S_2_ to the promoter. In this figure, an induction factor of *k_s_*/*b_s_* = 100 was assumed.

At a low concentration of activated regulator, *r_a_* = *r*_4_ = [R_2_S_2_], the production of signal molecules proceeds at a low baseline level, *b_s_*. At a higher concentration, the activated regulator is able to bind to the promoter site for the signal molecule with dissociation constant *K_s_*. The signal molecule production may therefore be expressed as:

(4) $$\kappa_{s}=\frac{K_{s}}{K_{s}+r_{a}}b_{s}+\frac{r_{a}}{K_{s}+r_{a}}k_{s}=\frac{\frac{b_{s}}{k_{s}}K_{s}+r_{a}}{K_{s}+r_{a}}k_{s}\approx \{\begin{array}{cc} b_{s} & ,r_{a}<\frac{b_{s}}{k_{s}}K_{s}\\ k_{s} & ,r_{a}>K_{s} \end{array}$$

normalized as produced number per time per intracellular volume. We invite you to examine Equation (4) and Figure 2. These describe an increase from the background production, *b_s_*, when *r_a_* is lower than $\frac{b_{s}}{k_{s}}K_{s}$, to the induced production, *k_s_*, when the concentration *r_a_* is above *K_s_*. In particular, note that the expected ignition point for feedback is at $r_{a}∼\frac{b_{s}}{k_{s}}K_{s}$, which need be at least an order of magnitude lower than that of *K_s_* to produce a significant effect. In Figure 2, we have assumed *k_s_* = 100*b_s_*.

## 5. The Size Measure

In the quasistatic limit of the reaction-diffusion equation, a simple dimensional analysis allows us to connect Equations (1), (2) and (4). Since the radius of the colony is the only size appearing in the problem, the Laplacian may be replaced by 1/ 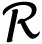
^2^. The signal molecule concentration is eliminated by inverting Equation (2) and inserting into Equation (1). Altogether, we arrive at:

(5) $$\sum \overset{\text{def}}{=}R^{2}\rho_{v}=\frac{2DK}{b_{s}}\underset{\text{feedback}}{\underset{︸}{\frac{b_{s}}{k_{s}}\frac{K_{s}+r_{a}}{\frac{b_{s}}{k_{s}}K_{s}+r_{a}}}}\underset{\text{forward}}{\underset{︸}{{\left(\frac{r_{a}}{r_{m}-r_{a}}\right)}^{\frac{1}{2}}}}$$

for cooperative second order ligand binding. Notably, we were able to collect all terms relating to size and geometry on the lhs and all terms relating to the intrinsic state of the cells on the rhs. This means that we can read the lhs as a proper size measure for the colony. The rhs is a nicely factorized form, where the “forward” corresponding to the standard accumulation of signal molecules as size or density increases has been separated from the influence of the positive “feedback”.

**Figure 3.**
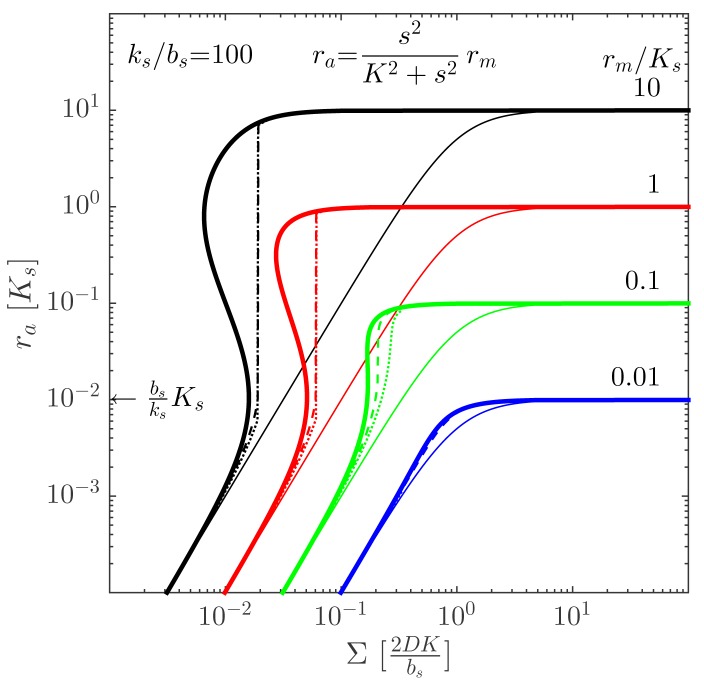
Intracellular concentration of activated regulator at the center of a bacterial colony as a function of the colony size. The signal molecule binding to the regulator is assumed second order and fully cooperative. Thin lines indicate the *r_a_* level without feedback. Dashed lines are the numerical solution with open boundary (Σ = 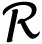
^2^*ρ_v_*), and dotted lines are with absorbing boundary ( $\sum =\frac{1}{3}R^{2}\rho_{v}$). Note that the ignition of the collective state always takes place at $r_{a}∼\frac{b_{s}}{k_{s}}K_{s}$, shown on the ordinate. Adapted from [11]—published by The Royal Society of Chemistry.

**Figure 4.**
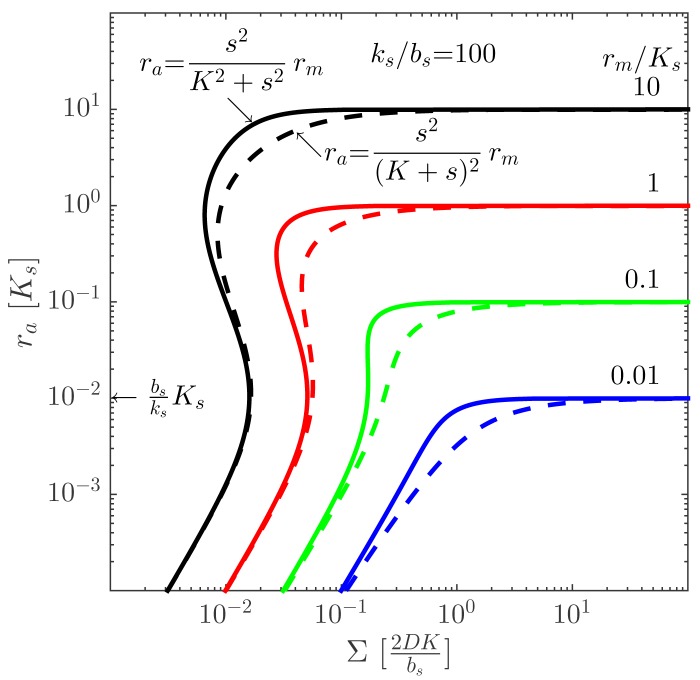
Intracellular concentration of the activated regulator at the center of a bacterial colony as a function of the colony size. The full curves represent cooperative ligand binding, and the dashed curves represent independent binding. When *r_m_* > *K_s_*, the hysteresis leading to a robust switch is present for cooperative ligand binding, as well as for independent binding. With cooperative binding, the switch is well defined at moderately lower *r_m_* values than obtained with non-cooperative binding. Adapted from [11]—published by The Royal Society of Chemistry.

This approximate form allows us to plot the activated regulator concentration, *r_a_* = [R_2_S_2_], as a function of the proper size, Σ = *ρ_v_* 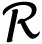
^2^, of the system. The resulting plot is shown in Figure 3 for different values of the maximal regulator concentration, *r_m_* = [R_2_S_2_]_max_. As expected, the ignition point is at $r_{a}=\frac{b_{s}}{k_{s}}K_{s}$. Provided that the maximal regulator concentration is sufficiently large, the quorum sensor produces a well-behaved, robust on/off switch when the size measure, Σ, gets sufficiently large. The size at which the ignition occurs may depend on the internal state of the cells, e.g., growth rate and temperature. The comparison between the switch with cooperative and non-cooperative ligand binding in Figure 4 shows that cooperativity extends the bi-stability region somewhat.

We refer to [11] for a detailed derivation of this result, including the factor “2” for open boundary conditions, which does not come out of the dimensional analysis given here. Other geometries and boundary conditions are elaborated, as well, and a detailed confirmation by numerical integration of the reaction-diffusion Equation (1) is carried out.

## 6. Signal Modulation in Regulator Production and Activation

The regulator has essentially two forms, the inactive form, where it cannot bind to the promoter for signal molecule production, and the active form, R_2_S_2_, which targets the promoter site with the effect of increasing the signal molecule production. As indicated in Figure 1b, the inactive form has sub-forms, the monomer, R, and the inactive regulator dimers, R_2_ and R_2_S. Reports indicate that the monomer form in the LuxRI homologues is susceptible to proteolytic degradation, while the dimer forms are resilient [12,15,16,21,22]. The combination of rapid proteolytic degradation of the regulator monomer with slow degradation of the dimeric forms brings two timescales into play and makes it possible for the quorum sensors to form a low-pass filter that may stabilize the switching to the collective mode. We will now take a look into this design as described in [12].

The signal sensor system in Figure 1b consists of an input channel and four regulatory units with concentrations *r*_1_ = [R], *r*_2_ = [R_2_], *r*_3_ = [R_2_S] and *r*_4_ = [R_2_S_2_]. The regulator formation and binding to the ligand is described by the kinetic equations:

(6) $$\frac{dr_{1}}{dt}=b_{1}R_{t}+2k_{2}^{-}r_{2}-2k_{2}^{+}r_{1}^{2}-\lambda_{1}r_{1}$$

(7) $$\frac{dr_{2}}{dt}=k_{2}^{+}r_{1}^{2}+k_{3}^{-}r_{3}-2k_{3}^{+}r_{2}s-\left(k_{2}^{-}+\lambda_{2}\right)r_{2}$$

(8) $$\frac{dr_{3}}{dt}=2k_{3}^{+}r_{2}s+2k_{4}^{-}r_{4}-k_{4}^{+}r_{3}s-\left(k_{3}^{-}+\lambda_{3}\right)r_{3}$$

(9) $$\frac{dr_{4}}{dt}=k_{4}^{+}r_{3}s-\left(2k_{4}^{-}+\lambda_{4}\right)r_{4}$$

The regulator is continuously produced at rate *b*_1_*R_t_* normalized to intracellular volume. On-rate constants are superscripted “+”, while off-rates are superscripted “−”. The dilution from cell growth is included in the proteolytic degradation, λ*_i_*, of each form of the regulator. The combinatorial factor two has been explicitly included, with this normalization *K*_3_ = *K*_4_ for non-cooperative ligand binding.

In comparison with the overall timescale set by the proteolytic degradation and the growth rate, the association and dissociation given in these equations can be regarded as fast. This results in the approximate quasistatic approximations $r_{1}^{2}=K_{2}r_{2}$, 2*r*_2_*s* = *K*_3_*r*_3_, and *r*_3_*s* = 2*K*_4_*r*_4_.

The slow timescale can be solved for by regarding the total regulator economy, which is derived by summing Equations (6), (7), (8), and (9):

(10) $$\frac{dr}{dt}=b_{1}R_{t}-\lambda r$$

(11) $$r=r_{1}+2r_{2}+2r_{3}+2r_{4}$$

(12) $$\lambda =\frac{r_{1}}{r}\lambda_{1}+\frac{2r_{2}}{r}\lambda_{2}+\frac{2r_{3}}{r}\lambda_{3}+\frac{2r_{4}}{r}\lambda_{4}$$

where the dimer contributions were counted twice, since they each contain two regulator units. These equations describe the relatively slow changes in total regulator concentration and are the key to understanding the design of the low-pass filtering to stabilize the quorum sensing switches. Note that 1/λ is the timescale for changing the total regulator concentration, and the steady-state concentration of the regulator is:

(13) $$r_{\text{steady state}}=\frac{b_{1}R_{t}}{\lambda }$$

To simplify, let us assume that all dimer forms are equally resilient to proteolytic degradation, *i.e.*, λ_2_ = λ_3_ = λ_4_ = *λ_d_*. For this illustrative calculation, let us assume cooperative binding of signal molecules to the dimerized regulator. We then have:

(14) $$\lambda =\frac{r_{1}}{r}\lambda_{1}+\frac{2r_{2}}{r}\lambda_{d}+\frac{2r_{4}}{r}\lambda_{d}$$

By changing the concentration of signal molecules, we can change the distribution between regulator forms rapidly, while changes in the total concentration of regulator will proceed slowly. This means that if, in the absence of S, we have *r*_1_*λ*_1_ ≫ 2*r*_2_*λ_d_*, we will get a slow response to a sudden increase in S concentration. This is effectively a low-pass filter. In line with the result for a first order system [13], the low-pass filtering is accompanied by a rescaling of the dissociation constant. The advantage of a low-pass filter in the activation process could be to make the quorum sensing switch robust to noise, even for very low values of the ignition threshold for the quorate state.

On the other hand, if we have *r*_1_*λ*_1_ ≪ 2*r*_2_*λ_d_*, we will get a fast response to an increase in S concentration. In the latter case, the introduction of signal molecules affects only the distribution between dimer forms, while the overall concentration of the regulator remains unchanged. In this limit, the introduction of signal molecules will not change *λ*, and we therefore get an all-pass filter and:

(15) $$r_{a}=r_{4}=\frac{b_{1}R_{t}}{2\lambda_{d}}\frac{s^{2}}{K^{2}+s^{2}}$$

with $K=\sqrt{K_{3}K_{4}}$ [12].

The low-pass and all-pass filter designs described here are analogous to the result derived for monomer binding by Garde *et al.* [13] and Claussen *et al.* [12]. The all-pass behavior was reported for a synthetic system where the natural promoter for regulator production was replaced by a more potent promoter on a plasmid with a high copy number, thereby suppressing the relative importance of the monomer [12]. However, the demonstration of a fast “degradation” of the activated regulator through the monomer channel provides evidence that the low-pass filtering is important in the LasRI system in *P. aeruginosa* [12,21]. The low-pass behavior has been reported for an effective first-order system [13]. The details of the filter designs remain a subject of ongoing investigations.

## 7. Discussion

We have presented a view of a biofilm as a smart material and demonstrated how a size-aware sensor system, capable of producing an on/off switch at a critical system size, can be modeled. The generic quorum sensor consists of only two genetic components, one component coding for the production of a diffusible signal molecule and one component coding for a regulator protein capable of binding the signal molecules. A positive feedback leads to a well-defined ignition point:

(16) $${\left[{\text{R}}_{2}{\text{S}}_{2}\right]}_{\text{ignition}}∼\frac{b_{s}}{k_{s}}K_{s}$$

for the quorate state. The dimerization of the regulator allows for flexibility between low-pass filtering and all-pass filtering in the binding of signal molecules to the regulator. We hypothesize that the low-pass filtering makes the system more robust to accidental ignition for systems operated at low signal molecule concentrations.

The positive feedback loop is effective when the induction factor for the feedback is much larger than the background production, *k_s_* ≫ *b_s_*. If this is the case and if growth conditions allow efficient binding of the regulator to the promoter site for signal molecule production, *r_m_* ≳ *K_s_*, the switch will ignite when the activated regulator concentration reaches the critical value.

The role of the quorum sensor system plays its primary role during micro-colony formation, while most systematic studies of constitutive promoters are conducted on planktonically growing cells [23–27]. There is a outstanding call for systematic studies of promoter activities in the quasi-stationary phase.

Typical biofilm thicknesses range from 13 to 60 μm mean (34 ± 10) μm in *Pseudomonas aeruginosa* [28]. Thicknesses in flow cells of mixed cultures of *Pseudomonas aeruginosa*, *Pseudomonas fluorescens*, *Klebsiella pneumoniae*, and *Stenotrophomonas maltophilia* range from (37 ±12) μm with flow to (51 ± 19) μm without flow [29] with corresponding horizontal dimension up to 500 μm. The order of magnitude in the size of biofilm structures is similar in *Staphylococcus aureus* (30−40 μm thick) [30], *Staphylococcus epidermidis* (240−590 μm diameter) [5]. It appears that the observed dimensions are in the high end for artificial conditions like implants or flow chambers whereas biofilm aggregates in chronic infections are in the lower end with an upper range limit of ∼200 μm [6].

An estimate of the geometrical size needed to ignite the quorate state may be obtained using the above expressions. At the ignition point, $r_{a}∼\frac{b_{s}}{k_{s}}K_{s}$, this results in

(17) $$\rho_{v}R^{2}∼\frac{DK}{b_{s}}\sqrt{\frac{\frac{b_{s}}{k_{s}}K_{s}}{r_{m}}}$$

for a functional switch. Estimates of parameters could be $\frac{b_{s}}{k_{s}}K_{s}∼0.001-0.01r_{m}$, *b_s_* ∼ 1000−10, 000nM/h [31], *K* ∼ 10−1000 nM [13,32], and *D* ∼ 1 mm^2^/h. This results in the very rough estimate

(18) R∼10μm−300μm

for a densely packed micro colony in which quorum sensing can be expected to play a role. If the colony is less densely packed the estimate is scaled accordingly. Notably, this is in qualitative agreement with the dimension of observed *in vivo* biofilm micro colonies.

As previously mentioned, the geometrical size and density at which the ignition occurs will depend on the internal state of the cells, e.g., growth rate and temperature. The size estimate given above may therefore vary, even within the same microbial strain. The complexity of the response of the biofilm to external conditions is further increased in, e.g., *P. aeruginosa*, where several quorum sensors are thought to be organized in a hierarchy, where the *rhl* system acts under the control of the *las* system [33,34].

## 8. Conclusions

We have reviewed the key properties of a single-loop quorum sensor with positive feedback and the main properties of the ligand binding. The quorum sensor changes the biofilm from a passive material to an active material capable of responding to external threats in accordance with the quorate state.

A proper measure for the size of the quorum sensing biofilm combines the geometry, cell density, and boundary conditions. When the maximal regulator concentration is sufficiently large to bind to the promoter for signal molecule production and the induction of the signal molecule production is at least an order of magnitude, a functioning quorum sensor with an all-or-none switch results.

## Glossary

**Parameter**	**Value**	

$k_{2}^{\pm }$		on/off rate constants for dimer formation
$k_{3}^{\pm }$, $k_{4}^{\pm }$		on/off rate constant for ligand binding
*K*_2_	$k_{2}^{-}/k_{2}^{+}$	dimer dissociation constant
*K*_3_,*K*_4_	$k_{3}^{-}/k_{3}^{+}$, $k_{4}^{-}/k_{4}^{+}$	ligand-dimer dissociation constants
*K*	$\sqrt{K_{3}K_{4}}$	dissociation constant for cooperative ligand binding
*b*_1_	∼1000h^−1^	production rate of R [26]
*b_s_*		background S production
*k_s_*	∼100 *b_s_*	maximal S production rate
*κ_s_*	*b_s_* < *κ_s_* < *k_s_*	S production
*λ*_1_	∼20h^−1^	R monomer degradation, [3]
*λ*_2_, *λ*_3_, *λ*_4_	<1 h^−1^	dimer degradation (including growth) [12,21]
*λ**_d_*		averaged dimer degradation rate
*R_t_*	[*P_R_*], [*P_S_*]	intracellular promoter density
*r*_1_, *r*_2_, *r*_3_, *r*_4_	[R], [R_2_], [R_2_S], [R_2_S_2_]	regulator monomer and dimer concentrations
*r*		total regulator concentration
*r_a_*	*r*_4_, [R_2_S_2_]	activated regulator concentration
*r_m_*	$\gg \frac{b_{s}}{k_{s}}K_{s}$	maximal *r_a_*
*s*	[S]	signal molecule concentration
*t*		time
*P_R_*		promoter for R synthesis
R		regulator protein
*P_S_*		promoter for S synthetase
S		signal molecule/ligand
*K*	10 nM−10 μm	R_2_S_2_ dissociation constant [13–15,32]
*K_s_*		R_2_S_2_-*P_S_* dissociation constant
*D*	∼2 mm^2^/h	diffusion constant for S (AHLs)
*ρ_v_*	<1	bacterial density (v/v)
Σ	*ρ_v_* ^2^	colony “size” measure
	10−70 μm	radius of colony [5,29]

## References

[1] Janga S.C., Collado-Vides J. (2007). Structure and evolution of gene regulatory networks in microbial genomes. Res. Microbiol..

[2] Fuqua W.C., Winans S.C., Greenberg E.P. (1994). Quorum sensing in bacteria: The LuxR-LuxI family of cell density-responsive transcriptional regulators. J. Bacteriol..

[3] Zhu J., Winans S.C. (1999). Autoinducer binding by the quorum-sensing regulator TraR increases affinity for target promoters *in vitro* and decreases TraR turnover rates in whole cells. Proc. Natl. Acad. Sci. USA.

[4] Nilsson P., Olofsson A., Fagerlind M., Fagerstrom T., Rice S., Kjelleberg S., Steinberg P. (2001). Kinetics of the AHL Regulatory System in a Model Biofilm System: How Many Bacteria Constitute a Quorum?. J. Mol. Biol..

[5] Fux C.A., Costerton J.W., Stewart PS., Stoodley P. (2005). Survival strategies of infectious biofilms. Trends Microbiol..

[6] Bjarnsholt T., Alhede M., Alhede M., Eickhardt-Sørensen S., Moser C., Kühl M., Jensen P., Høiby N. (2013). The *in vivo* biofilm. Trends Microbiol..

[7] Gonzalez J.E., Keshavan N.D. (2006). Messing with Bacterial Quorum Sensing. Microbiol. Mol. Biol. Rev..

[8] Bjarnsholt T., Ostrup Jensen P., Rasmussen T.B., Christophersen L., Calum H., Hentzer M., Hougen H.P., Rygaard J., Moser C., Eberl L. (2005). Pathogens and pathogenicity—Garlic blocks quorum sensing and promotes rapid clearing of pulmonary *Pseudomonas aeruginosa* infections. Microbiol. Read..

[9] Bjarnsholt T., Givskov M. (2007). The role of quorum sensing in the pathogenicity of the cunning aggressor Pseudomonas aeruginosa. Anal. Bioana. Chem..

[10] Alhede M., Bjarnsholt T., Jensen P., Phipps R., Moser C., Christophersen L., Christensen L., van Gennip M., Parsek M., Høiby N. (2009). Pseudomonas aeruginosa recognizes and responds aggressively to the presence of polymorphonuclear leukocytes. Microbiology.

[11] Ferkinghoff-Borg J., Sams T. (2014). Size of quorum sensing communities. Mol. BioSyst..

[12] Claussen A., Jakobsen T.H., Bjarnsholt T., Givskov M., Welch M., Ferkinghoff-Borg J., Sams T. (2013). Kinetic Model for Signal Binding to the Quorum Sensing Regulator LasR. Int. J. Mol. Sci..

[13] Garde C., Bjarnsholt T., Givskov M., Jakobsen T.H., Hentzer M., Claussen A., Sneppen K., Ferkinghoff-Borg J., Sams T. (2010). Quorum Sensing regulation in *Aeromonas hydrophila*. J. Mol. Biol..

[14] Welch M., Gross J., Hodgkinson J.T., Spring D.R., Sams T. (2013). Ligand binding kinetics of the quorum sensing regulator PqsR. Biochemistry.

[15] Welch M., Todd D.E., Whitehead N.A., McGowan S.J., Bycroft B.W., Salmond G.P. (2000). *N*-acyl homoserine lactone binding to the CarR receptor determines quorum-sensing specificity in *Erwinia*. EMBO J..

[16] Kiratisin P., Tucker K.D., Passador L. (2002). LasR, a Transcriptional Activator of *Pseudomonas aeruginosa* Virulence Genes, Functions as a Multimer. J. Bacteriol..

[17] Zhang R.G., Pappas T., Brace J.L., Miller P.C., Oulmassov T., Molyneaux J.M., Anderson J.C., Bashkin J.K., Winans S.C., Joachimiak A. (2002). Structure of a bacterial quorum-sensing transcription factor complexed with pheromone and DNA. Nature.

[18] Ventre I., Ledgham F., Prima V., Lazdunski A., Foglino M., Sturgis J.N. (2003). Dimerization of the quorum sensing regulator RhlR: Development of a method using EGFP fluorescence anisotropy. Mol. Microbiol..

[19] Schuster M., Urbanowski M.L., Greenberg E.P. (2004). Promoter Specificity in *Pseudomonas aeruginosa* Quorum Sensing Revealed by DNA Binding of Purified LasR. Proc. Natl. Acad. Sci. USA.

[20] Hill A.V. (1910). The possible effects of the aggregation of the molecules of haemoglobin on its dissociation curves. Proc. Physiol. Soc..

[21] Sappington K.J., Dandekar A.A., Oinuma K.I., Greenberg E.P. (2011). Reversible Signal Binding by the *Pseudomonas aeruginosa* Quorum-Sensing Signal Receptor LasR. MBio.

[22] Pinto U.M., Winans S.C. (2009). Dimerization of the quorum-sensing transcription factor TraR enhances resistance to cytoplasmic proteolysis. Mol. Microbiol..

[23] Bremer H., Dennis P.P., Neidhart F.C. (1996). Escherichia coli and Salmonella: Cellular and Molecular Biology.

[24] Liang S.T., Bipatnath M., Xu Y.C., Chen S.L., Dennis P., Ehrenberg M., Bremer H. (1999). Activities of constitutive promoters in Escherichia coli. J. Mol. Biol..

[25] Liang S.T., Xu Y.C., Dennis P., Bremer H. (2000). mRNA composition and control of bacterial gene expression. J. Bacteriol..

[26] Klumpp S., Zhang Z., Hwa T. (2009). Growth Rate-Dependent Global Effects on Gene Expression in Bacteria. Cell.

[27] Scott M., Gunderson C.W., Mateescu E.M., Zhang Z., Hwa T. (2010). Interdependence of cell growth and gene expression: Origins and consequences. Science.

[28] Stewart P.S., Peyton B.M., Drury W.J., Murga R. (1993). Quantitative observations of heterogeneities in Pseudomonas aeruginosa biofilms. Appl. Environ. Microbiol..

[29] Stoodley P., Lewandowski Z., Boyle J.D., Lappin-Scott H.M. (1999). Structural deformation of bacterial biofilms caused by short-term fluctuations in fluid shear: An in situ investigation of biofilm rheology. Biotechnol. Bioeng..

[30] Jefferson K.K., Goldmann D.A., Pier G.B. (2005). Use of confocal microscopy to analyze the rate of vancomycin penetration through Staphylococcus aureus biofilms. Antimicrob. Agents Chemother..

[31] Fagerlind M.G., Nilsson P., Harlén M., Karlsson S., Rice S.A., Kjelleberg S. (2005). Modeling the effect of acylated homoserine lactone antagonists in *Pseudomonas aeruginosa*. BioSystems.

[32] Andersen J.B., Sternberg C., Poulsen L.K., Bjorn S.P., Givskov M., Molin S. (1998). New Unstable Variants of Green Fluorescent Protein for Studies of Transient Gene Expression in Bacteria. Appl. Environ. Microbiol..

[33] Williams P., Stewart S., Camara M., Winson M., Chhabra S., Salmond G., Bycroft B. (1996). Signal transduction through quorum sensing in Pseudomonas aeruginosa. Pseudomonas: Molecular Biology and Biotechnology.

[34] Pesci E.C., Pearson J.P., Seed P.C., Iglewski B.H. (1997). Regulation of las and rhl quorum sensing in Pseudomonas aeruginosa. J. Bacteriol..

